# Functional modularity in lake-dwelling characin fishes of Mexico

**DOI:** 10.7717/peerj.3851

**Published:** 2017-09-22

**Authors:** Claudia Patricia Ornelas-García, Amando Bautista, Fabian Herder, Ignacio Doadrio

**Affiliations:** 1 Colección Nacional de Peces, Departamento de Zoología, Instituto de Biología, Universidad Nacional Autónoma de México, Mexico; 2 Centro Tlaxcala de Biología de la Conducta, Universidad Autónoma de Tlaxcala, Tlaxcala, Mexico; 3 Sektion Ichthyologie, Zoologisches Forschungsmuseum Alexander Koenig, Bonn, Germany; 4 Departamento de Biodiversidad y Biología Evolutiva, Museo Nacional de Ciencias Naturales, Madrid, Spain

**Keywords:** Modularity, Geometric morphometrics, *Astyanax*, Trophic diversity, Functional morphology

## Abstract

Modular evolution promotes evolutionary change, allowing independent variation across morphological units. Recent studies have shown that under contrasting ecological pressures, patterns of modularity could be related to divergent evolution. The main goal of the present study was to evaluate the presence of modular evolution in two sister lacustrine species, *Astyanax aeneus* and *A. caballeroi*, which are differentiated by their trophic habits. Two different datasets were analyzed: (1) skull X-rays from 73 specimens (35 *A. aeneus* and 38 *A. caballeroi*) to characterize skull variation patterns, considering both species and sex effects. For this dataset, three different modularity hypotheses were tested, previously supported in other lacustrine divergent species; (2) a complete body shape dataset was also tested for four modularity hypotheses, which included a total of 196 individuals (110 *Astyanax aeneus* and 86 *A. caballeroi*). Skull shape showed significant differences among species and sex (*P* < 0.001), where *Astyanax caballeroi* species showed an upwardly projected mandible and larger preorbital region. For the skull dataset, the modularity hypothesis ranked first included three partitioning modules. While for the complete body dataset the best ranked hypothesis included two modules (head vs the rest of the body), being significant only for *A. caballeroi*.

## Introduction

The study of morphological change is crucial to understanding biological diversity occurring in nature; however, evolutionary studies commonly deal with the consequences of the variability in populations, while less commonly dealing with its origins (Wagner & Altenberg, 1996).

Exploring morphological diversity raises questions of how morphological variation is organized in the organism, if morphological traits vary independently of each other, or if they are integrated, reflecting a coordination in their development, function and rate of change. In this respect, even though entire organisms must be functionally integrated, selection for functional performance could promote evolutionary changes in a modular manner (Cardini & Elton, 2008; Shirai & Marroig, 2010; Claverie & Patek, 2013; Klingenberg & Marugán-Lobón, 2013). A module refers to the occurrence of a set of phenotypic traits that are strongly integrated and that are relatively independent from other modules (Wagner & Altenberg, 1996; Klingenberg, 2008). Modular evolution can be considered as a fundamental aspect of morphological evolution (Cheverud, 1996; Wagner, 1996; Olson & Miller, 1999; Klingenberg, 2008), and studying modularity can improve understanding of genotype–phenotype maps in which there are few pleiotropic effects among traits (Wagner & Altenberg, 1996). In contrast with modularity, integration refers to the concentration of all variation in traits on a single dimension (Klingenberg, 2008).

Modularity is a variational property of any species, being variational in the potential of a trait to vary, while variability describes trait changes in response to environmental and genetic influences (Wagner & Altenberg, 1996). Modularity has been proposed as one of the key factors to explain morphological diversity in ray-finned fishes (Actinopterygians) (Larouche, Cloutier & Zelditch, 2015). Its effects could be associated with the exploitation of alternative resources in tropical (Cooper et al., 2010; Cooper et al., 2011) and temperate freshwater fish fauna (Wainwright, 1996; Kimmel et al., 2012).

Under ecological selective pressures, modular evolution may act as a promoter of morphological evolution (Kimmel et al., 2010; Kimmel et al., 2012; Makedonska, Wright & Strait, 2012). Particularly in ray-finned fishes, many changes in morphology have been associated with feeding and locomotion related to ecological divergence (Webb, 1982; Lauder & Liem, 1983; Webb, 1984; Webb & Weihs, 1986; Barluenga et al., 2006; Larouche, Cloutier & Zelditch, 2015; Magalhaes et al., 2015; Burress, Holcomb & Armbruster, 2016). Morphological diversity and modular organization are directly associated, either for feeding (Parsons, Cooper & Albertson, 2011) or locomotion (Larouche, Cloutier & Zelditch, 2015). Possibly one of the most well-known hypothesized cases of modular evolution associated with functional specialization and morphological reorganization is pharyngognathy in cichlids (among others; Liem, 1973); changes associated with processing food have been suggested as a key innovation driving bursts of diversification in this group. More recently, it has been shown that the occurrence of a separate module in the skull’s preorbital region in East African cichlids independently originated within Lakes Malawi, Victoria and Tanganyika, where speciation has been driven by trophic diversification (Cooper et al., 2010; Cooper et al., 2011).

Characiforms are a very interesting group in terms of their diversity, not only in species richness but also in morphology (Mirande, 2009; Oliveira et al., 2011). Previous studies have shown striking examples of trophic diversification and disparity in distinct groups (Sidlauskas, 2008). In this study, we aim to evaluate the role of modular evolution as a promoter of the morphological changes between closely related species, particularly under an ecological divergence scenario. Therefore, in our study we tested the presence of functional modules in two closely related species of characids, *Astyanax aeneus* and *Astyanax caballeroi*, which co-occur in Lake Catemaco (southern Mexico) and whose morphological differences are mainly related to alternative trophic niches (Contreras-Balderas & Rivera-Teillery, 1985; Miller, Minckley & Norris, 2005). The two species differ in tooth shape and dental formula and display significant differences in body depth, head shape and mouth orientation (Ornelas-García, Bastir & Doadrio, 2014). *A. caballeroi* has a fusiform body and has been suggested as a piscivorous fish (specialist), while the *A. aeneus* morpho species corresponds to an omnivorous (or generalist) fish (Miller, Minckley & Norris, 2005; Ornelas-García, Bastir & Doadrio, 2014). *A. aeneus* is a widely-distributed species (Bussing, 1985; Miller, Minckley & Norris, 2005; Ornelas-García, Domínguez-Domínuez & Doadrio, 2008), whereas *A. caballeroi* is endemic to Lake Catemaco (Contreras-Balderas & Rivera-Teillery, 1985). Despite the morphological differences previously described between these two lacustrine species, previous molecular studies have shown a lack of differentiation between them (Ornelas-García, Bastir & Doadrio, 2014). Thus, even if we decided to apply the current nomenclature for the species (Schmitter-Soto, 2016), it is possible that *A. caballeroi* corresponds to part of the phenotypic diversity of its sympatric species, *A. aeneus*.

The goal of our study was to determine if these two ecologically contrasting species (*A. aeneus* and *A. caballeroi*) inhabiting Lake Catemaco have evolved differential patterns of anatomical modularity. Hence, we examined alternative hypotheses of anatomical modules at the level of the skull and complete body. We expected a relationship between morphological diversity and modular organization, either for feeding or locomotion, to improve the alternative niche exploitation between closely related species, as has been suggested in other fish groups (Parsons, Cooper & Albertson, 2011).

Three specific hypotheses of skull modularity were considered: (1) the snout or preorbital (defined as “oral jaws and their supporting structures” Cooper et al., 2011; Parsons, Cooper & Albertson, 2011) vs the remainder of the skull (two modules); (2) preorbital region + eye + the remainder of the skull; and (3) snout (extended to include the suture between the frontal and parietal bones) + the remainder of the skull. The latter hypothesis was included to detect differences in the extent of a cranial hump that begins at the suture between the frontal and parietal bones, which is a feature that distinguishes the two species.

**Figure 1:**
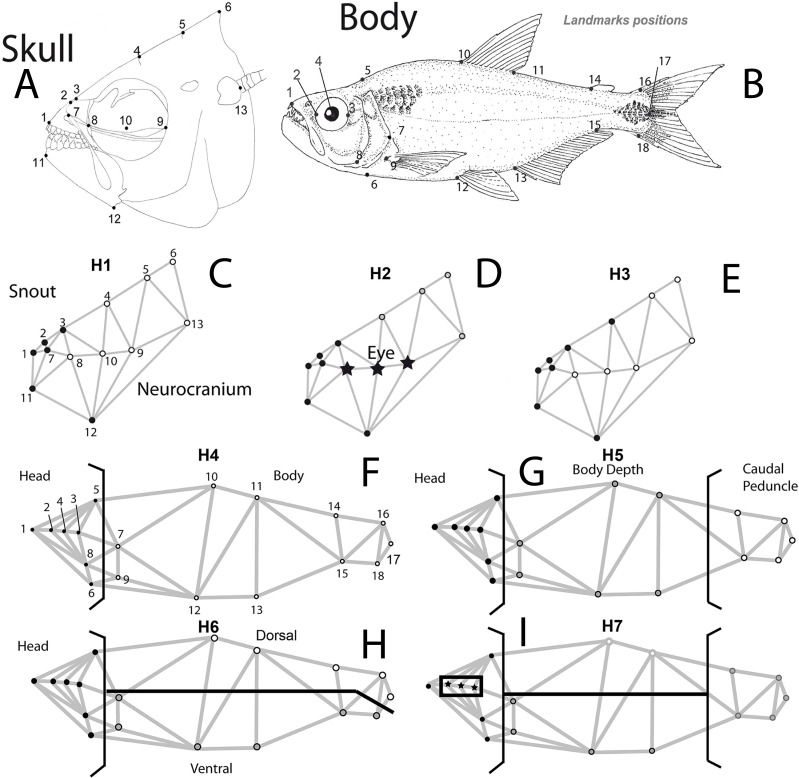
Body and skull landmark positions. Hypotheses of modularity for both data sets: (A) Skull landmarks positions and (B) body shape landmarks positions. (C) Hypothesis 1 (H1), snout or preorbital region (black dots) + the remainder of the skull (white dots), (D) Hypothesis 2 (H2), preorbital region (black dots) + eye (stars) + the remainder of the skull, (E) Hypothesis 3 (H3), snout (extended to include the suture between frontal and parietal bones) (black dots) + the remainder of the skull (white dots), (F) Hypothesis 4 (H4), head (black dots) + body (white dots), (G) Hypothesis 5 (H5), head (black dots) + central body (gray dots) + caudal peduncle (white dots), (H) Hypothesis 6 (H6), head (black dots) + dorsal (white dots) + ventral portions of the remainder of the remainder of the body (gray dots), (I) Hypothesis 7 (H7), eye (stars) + the remainder of the head (black dots) + dorsal and ventral portions of the central body and caudal peduncle (white and gray dots).

For the whole body, we tested the following modularity hypotheses: (4) head + body (a pattern that was previously tested in cichlids (Franchini et al., 2014)); (5) head + central body + caudal peduncle (Webb, 1982; Webb, 1984; Webb & Weihs, 1986); (6) head + dorsal + ventral portions of the remainder of the remainder of the body (Mullins et al., 1996; Nguyen et al., 1998); (7) eye + the remainder of the head + dorsal and ventral portions of the central body and caudal peduncle (Fig. 1).

## Materials and Methods

### Sample collection and taxonomic determination

**Figure 2:**
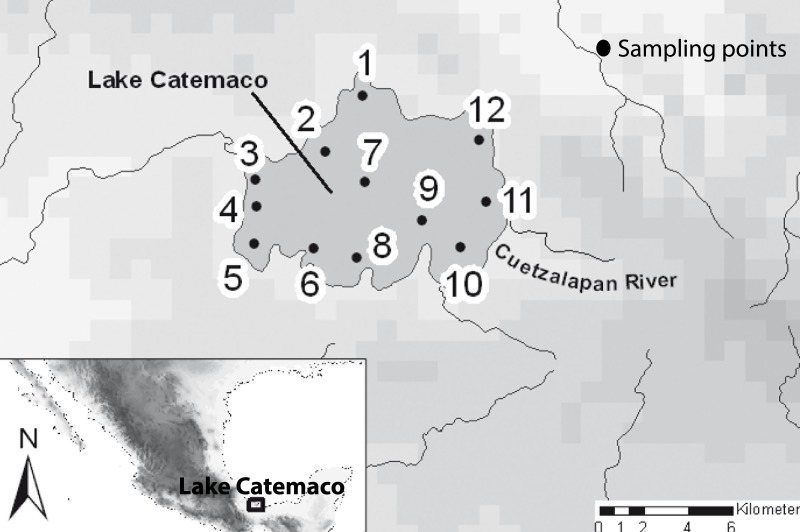
Map of the sampled localities in the Lake Catemaco. (1) Changos Island, (2) Agatepec Island, (3) Finca, (4) Maxacapan, (5) Victoria, (6) Pozolapan, (7) Center of the Lake, (8) Mimiahua, (9) Cuetzalapan Lago, (10) Margarita, (11) Cuetzalapan Río, and (12) Oxochapan.

A total of 11 sites within Lake Catemaco were sampled, including the different habitats present in the lake in terms of both depth and substrate (Fig. 2). In addition, we included samples from the Cuetzalapan river, which flows into Lake Catemaco. All sampling points were in the Sierra de Los Tuxtlas, Veracruz, southeastern Mexico. Sampling was carried out between 2008 and 2011, collecting a total of 196 individuals, using different diameter mesh-size nets. Voucher specimens were preserved in 95% ethanol and deposited in theMuseo Nacional de Ciencias Naturales of Madrid, Spain (MNCN). Fish were assigned to nominal species using diagnostic morphological characters (Contreras-Balderas & Rivera-Teillery, 1985; Miller, Minckley & Norris, 2005). Two datasets were obtained, for a subset of 73 individuals, we took lateral skull X-rays with the mandible fixed in occlusion (Supplemental Information 1), and for the complete body shape analyses, we included a total of 196 individuals preserved in ethanol and photographed in the same lateral orientation. The two morphs were categorized using their original species names, *A. aeneus*and *A. caballeroi* (Ornelas-García, Domínguez-Domínuez & Doadrio, 2008; Schmitter-Soto, 2016), and we also used a joint dataset combining both species (*A. aeneus* + *A. caballeroi*) for the modularity hypotheses.

### Data collection

To test skull shape differences between species, 13 homologous landmarks were digitized for the 73 X-ray images (Fig. 1, Table 1). Shape data were obtained using generalized Procrustes analysis (GPA) (Rohlf & Slice, 1990). To assess skull shape variation among species, a principal component analysis (PCA) and canonical variate analysis (CVA) (Zelditch et al., 2004; Dryden & Mardia, 2016) were performed using MorphoJ v. 1.05 software (Klingenberg, 2011). We tested for species-specific mean skull shape differences applying factorial ANOVA in MorphoJ v. 1.05 software (Klingenberg, Barluenga & Meyer, 2002; Klingenberg, 2011) to evaluate the main effects of both species and sex. To test modular hypotheses in the complete body, we analyzed a total of 196 lateral images for both species, with a total of 18 homologous landmarks (Fig. 1, Table 1), using the software TpsDig v.1.39 (Rohlf, 2003).

**Table 1:** List of landmarks used in the study.

No.	Landmark description	Type
Skull shape landmarks		
1	Anterior tip of the maxilla	LMK2
2	Anterior point of suture between premaxilla and ethmoid bone	LMK1
3	Suture between the ethmoid and the frontal bones	LMK1
4	Suture between the frontal and parietal bone	LMK1
5	Suture between the parietal and supraoccipital bones	LMK1
6	Supraoccipital crest	LMK1
7	Suture between the premaxillar and maxilla	LMK1
8	Anterior juxtaposition of the parasphenoid and ocular orbit	LMK1
9	Posterior limit of the ocular orbit	LMK1
10	Centre of the eye	LMK1
11	Anterior extent of the dentary	LMK2
12	Porterior extent of the dentary	LMK2
13	Posterior extreme of Atlas vertebrae	LMK1
Body shape landmarks		
1	Anterior tip of maxilla	LMK2
2	Anterior extent of orbit	LMK2
3	Posterior extent of orbit	LMK2
4	Centre of the orbit	LMK1
5	Posterior extent of supraoccipital	LMK1
6	Yugal	LMK1
7	Extent of the operculum	LMK2
8	Lower margin of preoperculum	LMK2
9	Dorsal insertion of the pectoral fin	LMK1
10	Anterior insertion of dorsal fin	LMK1
11	Posterior insertion of dorsal fin	LMK1
12	Anterior insertion of the pelvic fin	LMK1
13	Anterior insertion of anal fin	LMK1
14	Anteriodorsal insertion of adipose fin	LMK1
15	Posterior insertion of anal fin	LMK1
16	Dorsal junction of caudal fin and caudal peduncle	LMK1
17	Posterior extent of caudal peduncle	LMK1
18	Ventral junction of caudal fin and caudal peduncle	LMK1

### Functional modularity and allometry

To assess if modularity was present in the head of this lacustrine species pair, three different *a priori* modularity hypotheses were tested. These were based on functional, mechanical and anatomical criteria (Fig. 1). Additionally, from our joint dataset (196 individuals) for body shape, four modularity hypotheses were tested, considering different functional and developmental criteria (Fig. 1).

With morphometric data, the strength of covariation between different regions of a structure is used as a criterion to evaluate integration and modularity. A measure for quantifying covariation between sets of landmarks is therefore of critical importance. The RV-coefficient (Escoufier, 1973) provides an effective method for quantifying the strength of covariation (Klingenberg, 2009). Escoufier’s coefficient can be considered a multivariate extension of the expression for the correlation coefficient (*R*^2^) between two variables (Escoufier, 1973). It ranges from 0 to 1, with lower values of the RV—coefficient indicating lower covariation between modules (i.e., higher degree of modularity). The *P*-values correspond to the proportion of random partitions for which the RV-coefficient is less than or equal to the RV value for the partition of interest, and the null hypothesis corresponds to the random permutation of the landmarks into the different modules proposed, suggesting a complete independence between module sets (Klingenberg, 2009). The *M*_RV_ is used to compare more than two modules along the dataset (Fig. 1).

Since allometry and size could affect the patterns of integration, and therefore the identification of modularity (Klingenberg, 2009), we corrected for the possible effects of allometry in our datasets, computing the residuals from the regression of shape on centroid size (CS), and using those residuals in the different modularity hypotheses tested (Klingenberg, 2009; Klingenberg & Marugán-Lobón, 2013).

### Modularity hypotheses

To compare the different modularity hypotheses proposed, we followed the methodology suggested by Márquez (2008). We used the residuals from the regression of shape on size and the CS. All the analyses were carried out with the Mint software package (http://www-personal.umich.edu/ emarquez/morph/). We estimated a goodness of fit statistic, γ = gamma (Richtsmeier, Lele & Cole III, 2005). Models in which γ < 0 correspond to comparisons of observed covariances that are relatively low on average and hypothesized to be zero, and conversely, cases where γ > 0 occur when relatively high covariances are on average hypothesized to be zero. The best-fitting model is the one with the lowest γ value. Significance of γ for each hypothesis was obtained using a parametric Monte Carlo approach for each model and dataset (i.e., *A. aeneus*, *A. caballeroi* and joint datasets). A low *P*-value (*P* < 0.05) corresponds to large values of γ, indicating a large difference between data and the model, and thus a poorly fitting model. Confidence intervals for γ were obtained using a jackknife resampling method. Finally, the proportions of jackknife samples in which a model ranked first (i.e., had the lowest value of γ) was used to quantify jackknife support. In this analysis, the H0 is the null hypothesis corresponding to an integration among landmarks.

## Results

### Skull shape differences between species

**Figure 3:**
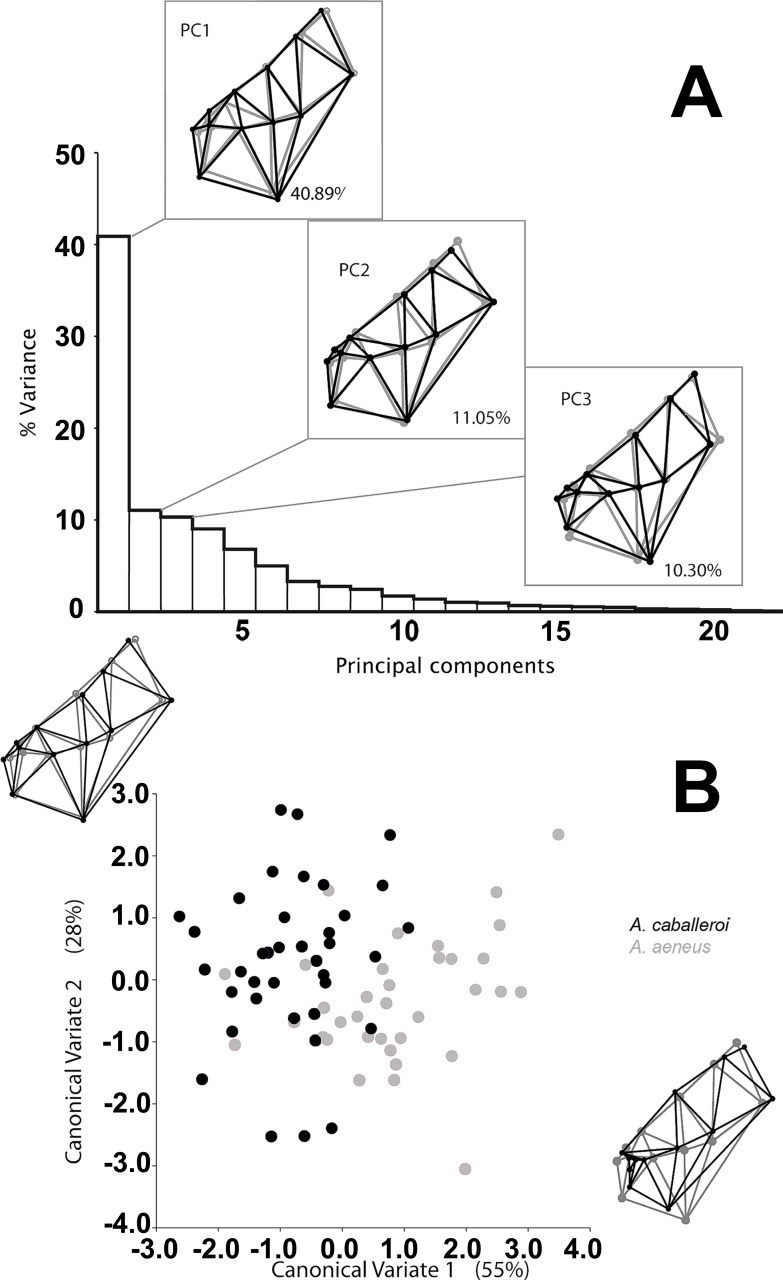
(A) Skull variation among species. The gray wireframe represents the centroid of the shape data and the black wireframe represents the positive deviation in shape along that axis. (B) Bivariate plot of CVA results from standardized Procrustes landmarks.

We analyzed a total of 73 skulls, 35 *A. aeneus* and 38 *A. caballeroi*, from eleven populations within Lake Catemaco, Mexico. We found significant differences in skull shape between species and sexes, while the interaction was not significant (Table 2). The main differences between the species were snout projection, eye size and head length; such differences could be identified in the wireframe from the PCA (Fig. 3). *A. caballeroi* presented a larger snout, corresponding to the wireframe of PC1, and accounting for 40% of the total variance, while the PC2 wireframe showed a difference among the most distal extreme in the supraoccipital crest (accounting for 11% of the variance). Finally, the PC3 wireframe showed differences along the axes accordingly to mouth position and the angle of dentary; this last PC accounts for 10% of the variance, giving a total of 60% of the variance accumulated in the first three components. The wireframe changes were positioned at the extremes of the CVA axes, where CV1 depicts an ordination based on the snout differences across the *x*-axis, while the CV2 corresponded to a combination between the lateral profile and snout elongation; as in the PCA, in the CVA, both axes were related to the preorbital region and head length. Point distribution in the canonical factors accumulated 80% of the variance, and even when we observed some important differences at the extremes of these coordinates, there was overlap between the two species (Fig. 3). Discriminant analysis showed significant differences between species (i.e., *A. aeneus vs. A. caballeroi*) based on Procrustes distances (*P*_*D*_ = 0.038 ±  < 0.00, *P* = 0.035).

**Table 2:** Factorial ANOVA statistics (Klingenberg, Barluenga & Meyer, 2002) results of skull shapes between the species.

Skull		Sum of squares	*df*	Mean square	*F*	*P*
CS	Species	291.41	1	291.41	64.16	0.0791
	Sex	116.32	1	116.32	25.61	0.124
	Species * sex	4.54	1	4.54	0.16	0.687
Shape	Species	0.0183	22	0.00083	6.25	<0.0001^**^
	Sex	0.0073	22	0.00033	2.23	0.0163^*^
	Species * sex	0.0028	22	0.00013	1.02	0.645

**Notes.**

CS, Centroid size.

* *P* > 0.05.

** *P* > 0.001.

### Integration in the skull variation

**Figure 4:**
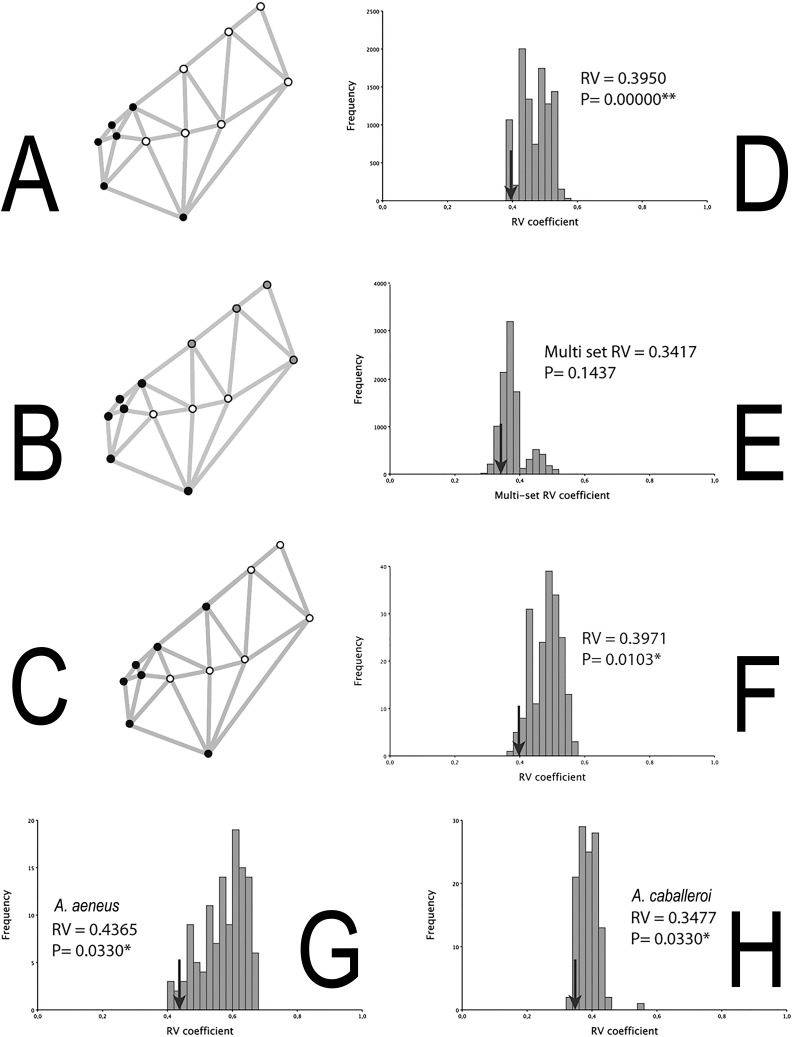
Skull modularity analyses. (A) H1, (B) H2, (C) H3, (D) Histogram representing the RV-coefficient for the H1 being tested for the combined data sets. (E) Histogram representing the RV-coefficient for the H2 being tested for the combined data sets. (F) Histogram representing the RV-coefficient for the H3 (snout *vs.* the remainder of the skull) being tested for the combined data sets. (G) Histogram representing RV-coefficients for the H3, for *A. aeneus* data set. (H) Histogram representing RV-coefficients for the H3, for *A. caballeroi* data set. Asterisks correspond to statistically significant models.

The integration histograms of *A. aeneus* and *A. caballeroi* skull hypotheses tend to be centered between 0.35 and 0.6 (Fig. 4). Among the modular hypotheses tested, using the RV-coefficients, two of three hypotheses showed significant signals of modular evolution (H1 and 3), while for the gamma values all the hypotheses were significant for the joint dataset (Table 3). H1 was significant in the joint dataset, but when we tested this hypothesis for each species independently, the *P*-values were non-significant in *A. aeneus* (*P* = 0.057, Table 3), while for *A. caballeroi* only gamma values were significant, although the RV-coefficient was very low (RV-coefficient = 0.34), and the species integration histograms were centered between 0.35 and 0.5 (Fig. 4, Table 3).

**Table 3:** Modularity hypotheses tested in *Astyanax* species.

Datasets	Rv or MRv	*P*	No. modules	γ	95% CI	*P* Monte Carlo	Jackknife support	Rank
**Skull**								
Joint datasets								
H1	0.395	<**0.001**	2	**−0.223**	[−0.23926, −0.19728]	1	97.90%	2
H2	0.3418	0.143	3	−***0.270***	[−0.29205, −0.23304]	1	100.00%	1
H3	0.3971	**0.010**	2	**−0.214**	[−0.23271, −0.18717]	1	97.90%	3
*Astyanax aeneus*								
H1	0.455	0.057	2	−0.232	[−0.25858, −0.19448]	0.576	100.00%	2
H2	0.3989	0.070	3	*−0.294*	[−0.33523, −0.23527]	0.250	100.00%	1
H3	0.4365	**0.033**	2	−0.216	[−0.24489, −0.17614]	0.497	100.00%	3
*A. caballeroi*								
H1	0.349095	0.066	2	−***0.189***	[−0.21407, −0.14864]	1	43.00%	2
H2	0.302051	0.510	3	*−0.189*	[−0.21416, −0.15116]	0.899	56.10%	1
H3	0.347791	**0.033**	2	**−0.182**	[−0.21371, −0.14043]	1	55.40%	3
**Whole Body**								
Joint datasets								
H1	0.422	0.271	2	***−0.238***	[−0.249, −0.224]	1	99.9%	1
H2	0.410	0.784	3	−0.199	[−0.207, −0.191]	1	35.7%	3
H3	0.331	0.121	3	−0.200	[−0.214, −0.187]	1	45.7%	2
H4	0.318	0.445	5	−0.194	[−0.210, −0.181]	1	82.9%	4
*Astyanax aeneus*								
H1	0.434	0.441	2	*−0.227*	[−0.244, −0.205]	1	88.9%	1
H2	0.441	0.859	3	−0.201	[−0.212, −0.188]	1	74.0%	4
H3	0.332	0.091	3	−0.213	[−0.227, −0.197]	1	77.3%	2
H4	0.329	0.464	5	−0.206	[−0.219, −0.189]	1	63.6%	3
*A. caballeroi*								
H1	0.353	0.132	2	***−0.311***	[−0.333, −0.284]	1	99.7%	1
H2	0.318	0.120	3	−0.250	[−0.275, −0.213]	1	61.2%	2
H3	0.331	0.235	3	**−0.165**	[−0.184, −0.135]	1	99.2%	4
H4	0.289	0.251	5	−0.246	[−0.290, −0.189]	1	60.4%	3

**Notes.**

The lowest gamma value is in cursives and the significant *P* values in gamma are in bold.

The two modules suggested in H3 (i.e., the snout *vs.* the remainder of the skull) differ from H1 in where the snout limit was defined (Fig. 1). In the former hypothesis, the limit was at the suture between the frontal and parietal bones (Landmark 5), while for H1 it was between the ethmoid and frontal bones. Even when the difference among the two hypotheses is very subtle (H1 and H3), this insertion point corresponds to one of the most conspicuous differences among the two species. H1 was not significant for *A. aeneus* but was significant for the gamma values in *A. caballeroi.* For both species, *A. aeneus* and *A. caballeroi*, as well as for the joint dataset, H3 showed significant RV-coefficient values (*P* ≤ 0.03, Table 3, Fig. 4B). For the gamma values of H3, only *A. aeneus* showed non-significant *P*-values.

### Whole body morphological integration

We analyzed a total of 196 individuals for the complete body, composed of 110 *A. aeneus* and 86 *A. caballeroi* (Supplemental Information 1). Different modularity hypotheses were supported among the species, presenting two significant gamma values for hypotheses 4 and 5 in *A. caballeroi*, while for *A. aeneus,* none of the hypotheses tested were significant (Table 3).

H4 considered two modules: head vs body (see Fig. 1A) and corresponded to a functional *a priori* hypothesis. Based on our results, this hypothesis showed the best gamma ranking, independent of the dataset used, and showed significant *P*-values for both the *A. caballeroi* and joint datasets (jackknife support > 99%, Table 3). Despite the low values of RV-coefficients in all datasets (closer to the lower extreme of the distribution), they were not significant (*P*-values > 0.05, Table 3).

**Figure 5:**
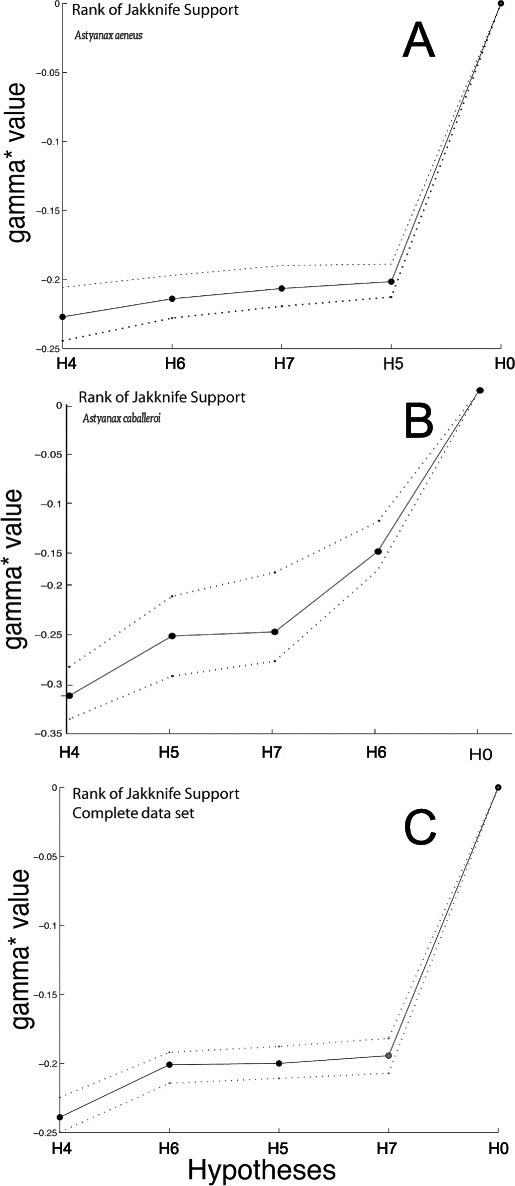
Graph with the Jackknife bootstrap ranking among the different modularity hypothesis being tested for the complete body dataset. (A) *Astyanax aeneus* Jackknife bootstrap ranking. (B) *Astyanax caballeroi* Jackknife bootstrap ranking. (C) Jackknife bootstrap ranking for the combined data sets.

H5, with three functional modules (Webb, 1982; Webb, 1984), showed no significant *P*-values in *M*_RV_-coefficient values nor gamma jackknifing (Table 3, Fig. 5). However, the analysis presented interesting gamma ranking patterns between the two species. For the *A. caballeroi* dataset H5 was the second best ranked, while for *A. aeneus* it was ranked at the fourth position. H5 considers the middle part of the body as an independent module to the caudal peduncle, and conspicuously, *A. caballeroi* (which represents a limnetic form) has a longer and narrower middle body compared to *A. aeneus*.

H6, with three tentative modules, head, dorsal and ventral regions forming different modules (Fig. 1A) (Mullins et al., 1996; Nguyen et al., 1998), showed significant jackknife support only for the *A. caballeroi* dataset (Table 3, Fig. 5), while it was not significant for *A. aeneus*.

Finally, H7 with five modules was non-significant for all the datasets tested (*P* > 0.05, Table 3). The H7 modules corresponded to the eye, head, and central body, while the caudal peduncle was subdivided into dorsal and ventral regions. This hypothesis was ranked in last place in the joint dataset and in third place for the other two datasets (*A. aeneus* and *A. caballeroi*).

### Modularity hypothesis comparisons among RV and gamma values

We found some differences in the best-fitting hypotheses across methods used, that is, between the Escoufier’s RV-coefficients and the standardized gamma statistics (Table 3). For the skull analyses, H2, with three modules (Fig. 1), was the best ranked among all the hypotheses tested, showing a 100% jackknife support for both joint and *A. aeneus* datasets. However, H2 did not recover any significant *P*-values for RV-coefficients; this hypothesis showed the lowest RV-coefficients for all data sets (0.341, 0.398 and 0.302 for the joint, *A. aeneus* and *A. caballeroi*, datasets, respectively (Table 3).

The top-ranked model using gamma values for the complete body analyses corresponded to H4, which includes two modules (Fig. 1, Table 3). This was the best model for all datasets (joint, *A. aeneus* and *A. caballeroi*), and jackknife support values for all datasets were 88.9%–99.9% (Table 3). Based on our results for the complete body, the *A. aeneus* dataset did not show significant results for any of the hypotheses, while *A. caballeroi* showed significant results only in the gamma values for hypotheses 4 and 6 but not for the RV or MRv-coefficients.

## Discussion

Modular evolution has been suggested as a variational property of an organism. The essence of the “building block hypothesis” is based on the suggestion that functional improvement in a trait can be achieved without compromising other optimized functions; therefore pleiotropic effects cannot be universal (Wagner & Altenberg, 1996). Because divergent adaptation of traits involved in different functions may be impeded by integration among parts, modularity offers a possible escape from such evolutionary constraint (Klingenberg, 2010). Within the vertebrates, Actinopterygians have demonstrated that modular organization can facilitate morphological diversification (Liem, 1973; Lauder & Liem, 1983; Cooper et al., 2010; Kimmel et al., 2010; Cooper et al., 2011; Parsons, Cooper & Albertson, 2011; Kimmel et al., 2012; Larouche, Cloutier & Zelditch, 2015; Parsons et al., 2016) in association with development (Kimmel et al., 2010; Kimmel et al., 2012), trophic diversification (Liem, 1973; Liem & Osse, 1975; Cooper et al., 2010; Cooper et al., 2011; Parsons, Cooper & Albertson, 2011; Parsons et al., 2016), and locomotor aspects (Larouche, Cloutier & Zelditch, 2015). Our study system gives a unique opportunity to explore the role of modularity in two closely related species, *A. aeneus* and *A. caballeroi*, which show morphological disparity in feeding (Miller, Minckley & Norris, 2005)—and locomotion—related traits (Ornelas-García, Bastir & Doadrio, 2014).

### Skull differences and modular organization

Our results support modularity in the preorbital region only for *A. caballeroi*, showing significant *P*-values for both gamma and RV-coefficients. Modularity in the preorbital region was previously suggested for multiple groups of African lacustrine cichlids and was mainly associated with exploitation of alternative resources (Cooper et al., 2010; Cooper et al., 2011). Other groups of characids (Sidlauskas, 2008) and minnows (Burress, Holcomb & Armbruster, 2016) have shown a marked morphological disparity associated with mouth position and snout length in relation to ecological diversification, suggesting a positive correlation between diversification rates and morphological shifts among minnows (Burress, Holcomb & Armbruster, 2016). Possibly one of the best-known hypotheses of modular evolution associated with trophic diversification is pharyngognathy in cichlids (among others; Liem, 1973), being associated with a burst of diversification in the group.

In our model system, the preorbital region (snout) in *A. aeneus* is characterized by being shorter than in *A. caballeroi*, particularly at the upper jaw, exhibiting a shorter premaxilla. Short oral jaws have been associated with powerful bites, which are advantageous for herbivores (Wainwright & Richard, 1995; Cooper et al., 2010). Previous studies have observed *Astyanax* species in tropical rivers to bite and tear plans and ingest terrestrial insects (Bonato, Burress & Fialho, 2017). By contrast, *A. caballeroi* has a larger oral jaw, a feature associated with the capture of large prey, and with an increased bite speed in other fish groups (Cooper et al., 2010). *Oligosarcus*, a genus of characiforms with similar trophic characteristics to *A. caballeroi* (i.e., longer lower jaws, greater snout length, longer tooth size and upturned mouth), has a piscivorous or carnivorous dietary pattern (Bonato, Burress & Fialho, 2017).

Morphological integration could originate from different mechanisms that produce joint variation. In this sense, environmental signals could affect the morphology and the mechanical forces through development. Those environmental signals are relevant to the phenotypic plasticity of the species, giving a flexibility in the developmental program in response to environmental stimuli as simple as temperature or as complex as diet (Klingenberg, 2010).

In this respect, skull modularity has been associated with ecological diversification processes not only in fish but also in other groups of vertebrates such as marsupials and lizards (Goswami, 2006; Goswami, 2007; Goswami & Polly, 2010). Further studies comparing the independent lineages of lacustrine sister species of *Astyanax* (Ornelas-García, Domínguez-Domínuez & Doadrio, 2008) could give additional evidence concerning the role of skull modularity in the ecological diversification of this group.

### Morphological integration and modularity in the body plan

For the complete body plan, we tested four *a priori* modular hypotheses based on both functional and developmental organization principles. We observed some differences in significance values for modularity hypotheses tested across the methods used, that is, between the RV-coefficients implemented in MorphoJ and gamma ranking implemented in Mint (Table 3). The major difference among these methods depends on the statistic used to infer the modules; the one using RV coefficients estimates the *P*-values based on the distribution simulated from random partitions (modules). The gamma ranking is based on goodness of fit derived from gamma values and a posterior jackknife test. The former considers the covariation between a subset of landmarks, while the latter treats each module as a multidimensional array throughout the entire analysis. There are also some important conceptual aspects to highlight between the different approaches used in our study, where the use of the standardized gamma statistics to assess the fit of models to covariance matrices of landmarks also implies evolvability, since modules are placed in orthogonal subspaces; therefore, a model will fit only in the modules that are independent of each other (Márquez, 2008). In contrast, this condition is not required for the Escoufier’s RV-coefficients, because modules delimited by the minimum Escoufier’s RV-coefficients between partitions can be highly integrated with each other so long as the intra-modular covariances are higher (Larouche, Cloutier & Zelditch, 2015). Despite these differences, some major patterns were coincident between the two methodologies applied.

In general terms, and similarly to the skull data set, *A. caballeroi* exhibited a higher number of significant modularity hypotheses than *A. aeneus* (Table 3, Fig. 5). Among all hypotheses tested, hypothesis 4 showed the lowest gamma values, and it was ranked first (gamma value = − 0.29), independently of the dataset used: *A. aeneus*, *A. caballeroi* or the joint dataset. In hypothesis 4, the body was partitioned into two modules: head *vs* the rest of the body, and based on a functional perspective, the occurrence of these modules strongly affects the trophic and swimming performance of the fish (Webb, 1982). This result agrees with our previous morphological studies, as the most striking differences between the two species correspond to the head morphology and body height (Ornelas-García, Bastir & Doadrio, 2014). Here we find evidence for a possible link between morphological differentiation and a functional process, which we propose could be related to the occurrence of modular evolution between these two species.

Similar to the previous hypothesis, hypothesis 5 was based on functional principles, considering three functional modules: head, central body and caudal regions (Fig. 5). The modules from this hypothesis could be linked to a differential habitat use, where the head is correlated to ecological variables (such as type of substrate) and food acquisition (Bouton, Visser & Barel, 2002; Jenkins & Burkhead, 1993; Liem, 1980; Wainwright & Richard, 1995), while the central body and caudal peduncle are related to swimming speed and maneuverability (Martinez et al., 2003; Plaut, 2000; Walker, 1997; Wardle, Videler & Altringham, 1995; Webb, 1984). In this hypothesis, we found different results depending on the species analyzed (Table 3). In *A. caballeroi*, hypothesis 5 was ranked second (*M*_RV_ = 0.318, *P* > 0.05), but it was fourth in *A. aeneus* (*M*_RV_ = 0.410, *P* > 0.05; Fig. 5). In this regard, in our previous morphological study we suggested *A. caballeroi* as a limnetic form, with a fusiform body (lower body depth) and with a slender peduncle (Ornelas-García, Bastir & Doadrio, 2014), likely associated with living in open waters and sustaining high swimming speeds (Langerhans & Reznick, 2010). Slender peduncles have been associated in other fish groups with trophic habits, mainly predation (Skulason & Smith, 1995; Walker, 1997). The mentioned morphological differences in the specialized *A. caballeroi* could be the main reason for the modularity differences between both species.

Hypothesis 6 was the second best ranked for both the *A. aeneus* and joint datasets. This hypothesis partitioned the body into three modules: head, ventral and dorsal regions (Table 3, Fig. 5). Previous studies have found that there is a genetic basis for the occurrence of dorsal and ventral modules, defined from dorso-ventral deformities in zebrafish (i.e., the genes *minifin*, *piggytail*, *swirl*, *somitabun*, *lost-a-fin* and *snailhouse*) (Mullins et al., 1996; Nguyen et al., 1998). However, further research is needed to better understand the reasons for this partitioning in the generalist species and to test its presence in other characiforms.

### Modular evolution in the trophic-specialized characid system

The study of morphological evolution is essential to understanding the potential effect of a novelty in response to ecological pressures (Wagner, 1996). Trophic niche specialization could be the result of a trait modification (e.g., teeth shape, gill-raker fusion or number variation), which could act as the promoter of diversification under differential ecological conditions. One of the most striking examples is in lacustrine fish radiations (Liem & Osse, 1975; Schluter, 1993; Schluter, 1996; Barluenga & Meyer, 2004; Herder & Freyhof, 2006; Salzburger, 2009; Cooper et al., 2010; Cooper et al., 2011; Pfaender et al., 2011; Muschick, Indermaur & Salzburger, 2012).

The genus *Astyanax* represents a very conspicuous model system to study morphological evolution driven by ecological divergence, with the most extreme phenotype being one adapted to a troglobitic environment (Gross, 2016). However, less attention has been given to the lacustrine species pairs of *Astyanax*, which have evolved in a convergent manner during different episodes of the evolutionary history of the genus in Mesoamerica (Ornelas-García, Domínguez-Domínuez & Doadrio, 2008).

The present study demonstrates evidence of modular evolution, opening further questions about the presence of these modules in other pairs of lacustrine species within the *Astyanax* genus. In this respect, the preorbital module in the skull dataset, as well as the partitioning of the body into two modules (head and remaining body), were the two best ranked hypotheses tested among the *Astyanax* species in this study, suggesting that modularity could have a role in trophic and locomotor segregation, similar to other freshwater groups (Cooper et al., 2010; Cooper et al., 2011; Larouche, Cloutier & Zelditch, 2015). Further studies including more lacustrine species complexes within the *Astyanax* genus may shed light on the patterns and implications of modularity in specialized trophic systems.

##  Supplemental Information

10.7717/peerj.3851/supp-1 Supplemental Information 1 List of individuals used in the morphometric for both datasets: whole body and skulls

10.7717/peerj.3851/supp-2 Supplemental Information 2 Schematic representation of the role of modular evolution under an adaptive divergence scenario Based on this diagram we suggested a reciprocal interaction among trophic, morphological, modular and genetic divergence, which could facilitate the adaptive divergence.

10.7717/peerj.3851/supp-3 Supplemental Information 3 Table with the raw data used in the study

## Additional Information and Declarations

### Data Availability

The following information was supplied regarding data availability: Fish vouchers were deposited at the Museo Nacional de Ciencias Naturales of Madrid, Spain (MNCN).
