# Supplementary material for: Functional modularity in lake-dwelling characin fishes of Mexico
Source: PeerJ. 2017 Sep 22;5:e3851. doi: 10.7717/peerj.3851 (PMC5611896; doi:10.7717/peerj.3851)

Functional modularity in lake-dwelling characin fishes of Mexico

Claudia Patricia Ornelas – García[§]; Amando Bautista; Fabian Herder; and Ignacio Doadrio

patriciaornelasg@gmail.com

Individual	Population	Morphotype	SEX	External morphology	Skull
Aga_1	Isla Agatepec	*A. caballeroi*	F	X	X
Aga_10	Isla Agatepec	*A. aeneus*	J	X	X
Aga_14	Isla Agatepec	*A. aeneus*	M	X	X
Aga_3	Isla Agatepec	*A. caballeroi*	M	X	
Aga_5	Isla Agatepec	*A. aeneus*	F	X	X
Aga_8	Isla Agatepec	*A. aeneus*	M	X	
Cate_1	Catemaco Center	*A. caballeroi*	F	X	X
Cate_10	Catemaco Center	*A. caballeroi*	F	X	X
Cate_11	Catemaco Center	*A. caballeroi*	F	X	
Cate_13	Catemaco Center	*A. aeneus*	F	X	X
Cate_15	Catemaco Center	*A. aeneus*	F	X	
Cate_17	Catemaco Center	*A. caballeroi*	F	X	
Cate_18	Catemaco Center	*A. caballeroi*	F	X	X
Cate_19	Catemaco Center	*A. aeneus*	F	X	X
Cate_2	Catemaco Center	*A. caballeroi*	M	X	
Cate_20	Catemaco Center	*A. caballeroi*	M	X	
Cate_21	Catemaco Center	*A. caballeroi*	F	X	
Cate_22	Catemaco Center	*A. caballeroi*	F	X	
Cate_23	Catemaco Center	*A. caballeroi*	M	X	
Cate_24	Catemaco Center	*A. caballeroi*		X	
Cate_25	Catemaco Center	*A. caballeroi*	M	X	
Cate_26	Catemaco Center	*A. caballeroi*	M	X	
Cate_27	Catemaco Center	*A. caballeroi*		X	X
Cate_28	Catemaco Center	*A. caballeroi*	F	X	
Cate_29	Catemaco Center	*A. caballeroi*	M	X	X
Cate_3	Catemaco Center	*A. caballeroi*	M	X	
Cate_31	Catemaco Center	*A. aeneus*	F	X	X
Cate_32	Catemaco Center	*A. aeneus*	F	X	
Cate_37	Catemaco Center	*A. aeneus*	F	X	
Cate_4	Catemaco Center	*A. caballeroi*	F	X	
Cate_5	Catemaco Center	*A. caballeroi*	F	X	X
Cate_6	Catemaco Center	*A. caballeroi*	F	X	
Cate_7	Catemaco Center	*A. caballeroi*	F	X	
Cate_8	Catemaco Center	*A. caballeroi*	F	X	
Cate_9	Catemaco Center	*A. caballeroi*	M	X	

Cha_14	Isla Changos	*A. aeneus*	M	X	X
Cha_16	Isla Changos	*A. aeneus*	J	X	
Cha_17	Isla Changos	*A. aeneus*	F	X	
Cha_2	Isla Changos	*A. aeneus*	M	X	X
Cha_20	Isla Changos	*A. aeneus*	M	X	X
Cha_36	Isla Changos	*A. aeneus*	M	X	X
CueL_1	Cuetzalapan Lago	*A. aeneus*	F	X	
CueL_10	Cuetzalapan Lago	*A. aeneus*	F	X	X
CueL_11	Cuetzalapan Lago	*A. aeneus*	M	X	X
CueL_12	Cuetzalapan Lago	*A. aeneus*	F	X	X
CueL_13	Cuetzalapan Lago	*A. caballeroi*	M	X	
CueL_14	Cuetzalapan Lago	*A. aeneus*	F	X	
CueL_16	Cuetzalapan Lago	*A. aeneus*	F	X	X
CueL_17	Cuetzalapan Lago	*A. aeneus*	F	X	X
CueL_18	Cuetzalapan Lago	*A. aeneus*	M	X	
CueL_19	Cuetzalapan Lago	*A. aeneus*	M	X	X
CueL_23	Cuetzalapan Lago	*A. caballeroi*	F	X	X
CueL_24	Cuetzalapan Lago	*A. caballeroi*	F	X	X
CueL_3	Cuetzalapan Lago	*A. aeneus*	J	X	
CueL_7	Cuetzalapan Lago	*A. aeneus*	F	X	
CueR_1	Cuetzalapan Río	*A. aeneus*	F	X	X
CueR_11	Cuetzalapan Río	*A. aeneus*	M	X	X
CueR_12	Cuetzalapan Río	*A. aeneus*	M	X	X
CueR_13	Cuetzalapan Río	*A. aeneus*	M	X	
CueR_14	Cuetzalapan Río	*A. caballeroi*	F	X	X
CueR_15	Cuetzalapan Río	*A. caballeroi*	M	X	
CueR_16	Cuetzalapan Río	*A. caballeroi*		X	X
CueR_18	Cuetzalapan Río	*A. caballeroi*	M	X	X
CueR_20	Cuetzalapan Río	*A. aeneus*	M	X	
CueR_21	Cuetzalapan Río	*A. aeneus*	F	X	X
CueR_22	Cuetzalapan Río	*A. aeneus*	F	X	X
CueR_23	Cuetzalapan Río	*A. aeneus*	M	X	X
CueR_25	Cuetzalapan Río	*A. caballeroi*	M	X	
CueR_26	Cuetzalapan Río	*A. caballeroi*	M	X	X
CueR_27	Cuetzalapan Río	*A. aeneus*	F	X	X
CueR_3	Cuetzalapan Río	*A. aeneus*	M	X	
CueR_4	Cuetzalapan Río	*A. aeneus*	M	X	
CueR_6	Cuetzalapan Río	*A. aeneus*	M	X	
CueR_7	Cuetzalapan Río	*A. aeneus*	F	X	X
CueR_9	Cuetzalapan Río	*A. aeneus*	F	X	
Finca_1	La Finca	*A. aeneus*	M	X	
Finca_11	La Finca	*A. aeneus*	M	X	

Finca_13	La Finca	*A. caballeroi*	F	X	
Finca_15	La Finca	*A. aeneus*	M	X	
Finca_16	La Finca	*A. aeneus*	F	X	
Finca_17	La Finca	*A. aeneus*	F	X	
Finca_2	La Finca	*A. aeneus*	M	X	
Finca_3	La Finca	*A. aeneus*	M	X	
Finca_5	La Finca	*A. aeneus*	M	X	
Finca_7	La Finca	*A. aeneus*	F	X	
Finca_8	La Finca	*A. aeneus*	F	X	
Finca1_10	La Finca	*A. caballeroi*	F	X	
Finca1_11	La Finca	*A. aeneus*	F	X	
Finca1_14	La Finca	*A. aeneus*	F	X	
Finca1_17	La Finca	*A. caballeroi*	M	X	
Finca1_18	La Finca	*A. caballeroi*	M	X	
Finca1_2	La Finca	*A. aeneus*	F	X	
Finca1_20	La Finca	*A. aeneus*	F	X	
Finca1_21	La Finca	*A. caballeroi*	F	X	
Finca1_22	La Finca	*A. caballeroi*	F	X	
Finca1_23	La Finca	*A. aeneus*	M	X	
Finca1_24	La Finca	*A. caballeroi*	F	X	
Finca1_25	La Finca	*A. aeneus*	F	X	
Finca1_3	La Finca	*A. caballeroi*	F	X	
Finca1_4	La Finca	*A. aeneus*	M	X	
Finca1_5	La Finca	*A. aeneus*	F	X	
Finca1_6	La Finca	*A. aeneus*	F	X	
Finca1_7	La Finca	*A. aeneus*	F	X	
Mar_1	Margaritas	*A. aeneus*	M	X	X
Mar_12	Margaritas	*A. aeneus*	M	X	
Mar_13	Margaritas	*A. caballeroi*	F	X	
Mar_14	Margaritas	*A. caballeroi*	F	X	X
Mar_2	Margaritas	*A. caballeroi*	F	X	X
Mar_3	Margaritas	*A. caballeroi*	F	X	
Mar_4	Margaritas	*A. caballeroi*	F	X	X
Mar_5	Margaritas	*A. aeneus*	M	X	
Mar_7	Margaritas	*A. caballeroi*	F	X	X
Maxa_10	Maxacapan	*A. caballeroi*	F	X	
Maxa_11	Maxacapan	*A. caballeroi*	F	X	
Maxa_12	Maxacapan	*A. caballeroi*		X	
Maxa_13	Maxacapan	*A. caballeroi*	F	X	X
Maxa_14	Maxacapan	*A. caballeroi*	M	X	
Maxa_2	Maxacapan	*A. aeneus*	F	X	
Maxa_3	Maxacapan	*A. aeneus*	F	X	

Maxa_4	Maxacapan	*A. caballeroi*	J	X	
Maxa_5	Maxacapan	*A. caballeroi*	F	X	
Maxa_6	Maxacapan	*A. caballeroi*	M	X	
Maxa_7	Maxacapan	*A. caballeroi*	F	X	
Maxa_8	Maxacapan	*A. caballeroi*	F	X	
Maxa_9	Maxacapan	*A. caballeroi*	M	X	
Mimi_1	Mimiagua	*A. aeneus*	M	X	X
Mimi_10	Mimiagua	*A. caballeroi*	M	X	X
Mimi_11	Mimiagua	*A. aeneus*	F	X	
Mimi_13	Mimiagua	*A. aeneus*	M	X	X
Mimi_14	Mimiagua	*A. aeneus*	M	X	
Mimi_2	Mimiagua	*A. aeneus*	M	X	
Mimi_4	Mimiagua	*A. aeneus*	M	X	
Mimi_5	Mimiagua	*A. aeneus*	F	X	
Mimi_6	Mimiagua	*A. aeneus*	F	X	
Mimi_7	Mimiagua	*A. aeneus*	M	X	
Mimi_9	Mimiagua	*A. caballeroi*	M	X	X
Oxo_10	Oxochapan	*A. aeneus*	M	X	
Oxo_11	Oxochapan	*A. aeneus*	F	X	
Oxo_12	Oxochapan	*A. aeneus*	F	X	
Oxo_14	Oxochapan	*A. aeneus*	M	X	
Oxo_16	Oxochapan	*A. aeneus*	F	X	
Oxo_18	Oxochapan	*A. caballeroi*	F	X	
Oxo_21	Oxochapan	*A. aeneus*	M	X	
Oxo_22	Oxochapan	*A. aeneus*	F	X	
Oxo_26	Oxochapan	*A. aeneus*	M	X	
Oxo_28	Oxochapan	*A. aeneus*	F	X	
Oxo_29	Oxochapan	*A. aeneus*	M	X	
Oxo_3	Oxochapan	*A. aeneus*	J	X	
Oxo_31	Oxochapan	*A. aeneus*	F	X	
Oxo_4	Oxochapan	*A. aeneus*	F	X	
Oxo_5	Oxochapan	*A. aeneus*	J	X	
Oxo_7	Oxochapan	*A. aeneus*	M	X	
Oxo_8	Oxochapan	*A. aeneus*	F	X	
Oxo_9	Oxochapan	*A. aeneus*	F	X	
Pozo_1	Pozolapan	*A. aeneus*	M	X	
Pozo_11	Pozolapan	*A. caballeroi*	M	X	
Pozo_12	Pozolapan	*A. caballeroi*	M	X	
Pozo_13	Pozolapan	*A. caballeroi*	F	X	
Pozo_14	Pozolapan	*A. aeneus*	M	X	
Pozo_15	Pozolapan	*A. aeneus*	F	X	
Pozo_18	Pozolapan	*A. aeneus*	M	X	

Pozo_2	Pozolapan	*A. caballeroi*	M	X	
Pozo_20	Pozolapan	*A. aeneus*	M	X	
Pozo_23	Pozolapan	*A. aeneus*	F	X	
Pozo_24	Pozolapan	*A. aeneus*	M	X	
Pozo_25	Pozolapan	*A. aeneus*	M	X	
Pozo_26	Pozolapan	*A. aeneus*	M	X	
Pozo_3	Pozolapan	*A. caballeroi*	F	X	
Pozo_5	Pozolapan	*A. caballeroi*	M	X	
Pozo_6	Pozolapan	*A. aeneus*	M	X	
Pozo_7	Pozolapan	*A. aeneus*	F	X	
Vic_1	La Victoria	*A. caballeroi*	F	X	
Vic_10	La Victoria	*A. aeneus*	M	X	X
Vic_11	La Victoria	*A. caballeroi*	F	X	X
Vic_12	La Victoria	*A. caballeroi*	F	X	X
Vic_13	La Victoria	*A. caballeroi*	F	X	X
Vic_14	La Victoria	*A. caballeroi*	F	X	X
Vic_15	La Victoria	*A. caballeroi*	F	X	X
Vic_16	La Victoria	*A. aeneus*	F	X	X
Vic_17	La Victoria	*A. aeneus*	F	X	X
Vic_19	La Victoria	*A. caballeroi*	F	X	X
Vic_2	La Victoria	*A. caballeroi*	F	X	X
Vic_21	La Victoria	*A. caballeroi*	F	X	X
Vic_23	La Victoria	*A. caballeroi*	M	X	X
Vic_24	La Victoria	*A. caballeroi*	F	X	X
Vic_25	La Victoria	*A. caballeroi*	F	X	X
Vic_26	La Victoria	*A. caballeroi*	M	X	X
Vic_27	La Victoria	*A. caballeroi*	F	X	X
Vic_28	La Victoria	*A. caballeroi*	F	X	X
Vic_29	La Victoria	*A. caballeroi*	F	X	X
Vic_3	La Victoria	*A. caballeroi*	F	X	X
Vic_4	La Victoria	*A. aeneus*	F	X	X
Vic_5	La Victoria	*A. aeneus*	F	X	X
Vic_6	La Victoria	*A. aeneus*	F	X	X
Vic_8	La Victoria	*A. aeneus*	F	X	X
Vic_9R	La Victoria	*A. caballeroi*	F	X	X

J=juvenile, F=femal and M= male

Supporting Material 2.

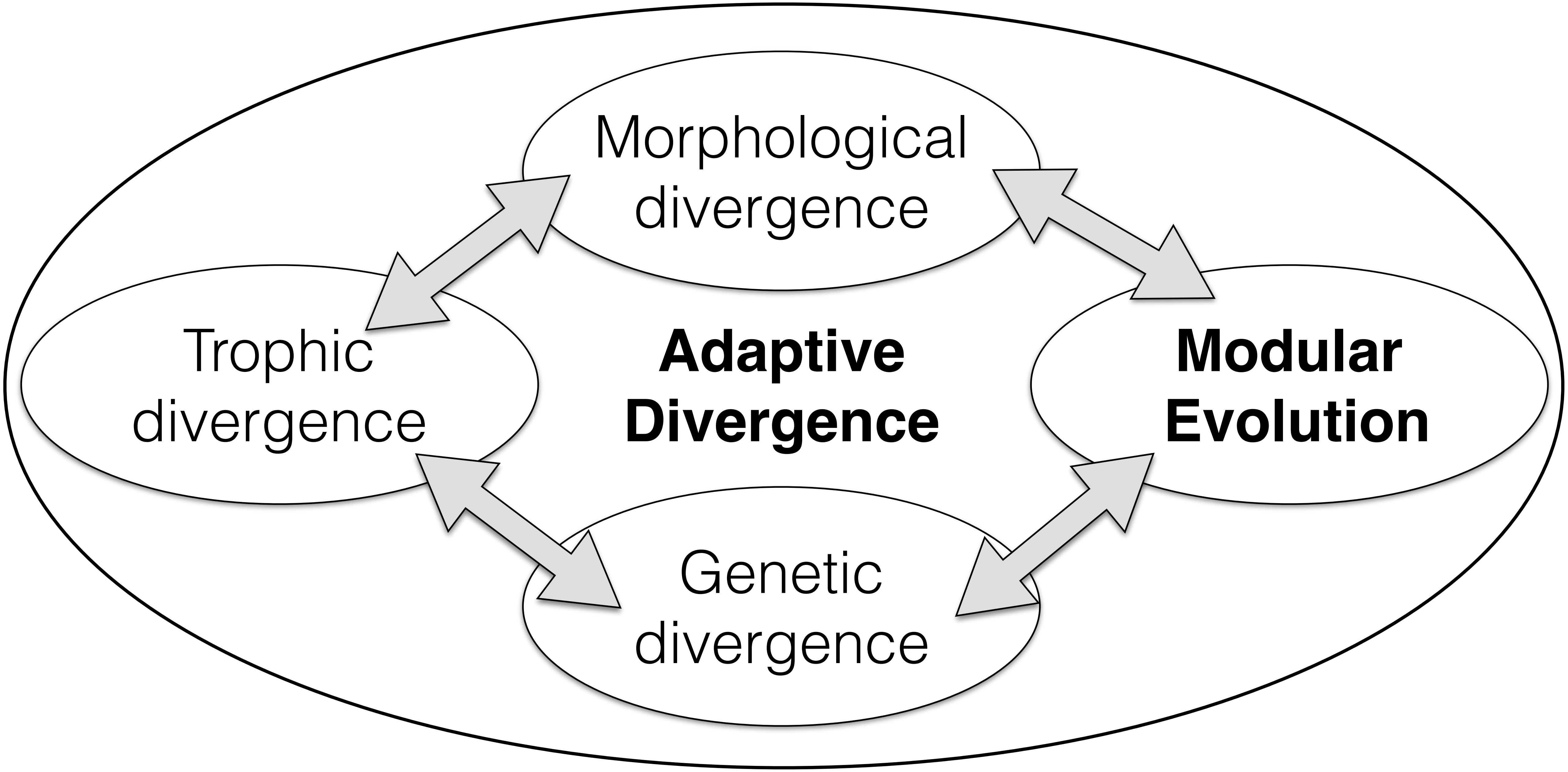

Supplement: Supplemental Information 1 [file peerj-05-3851-s001.pdf]
