# Supplementary figures and images for: Functional modularity in lake-dwelling characin fishes of Mexico
Source: PeerJ. 2017 Sep 22;5:e3851. doi: 10.7717/peerj.3851 (PMC5611896; doi:10.7717/peerj.3851)

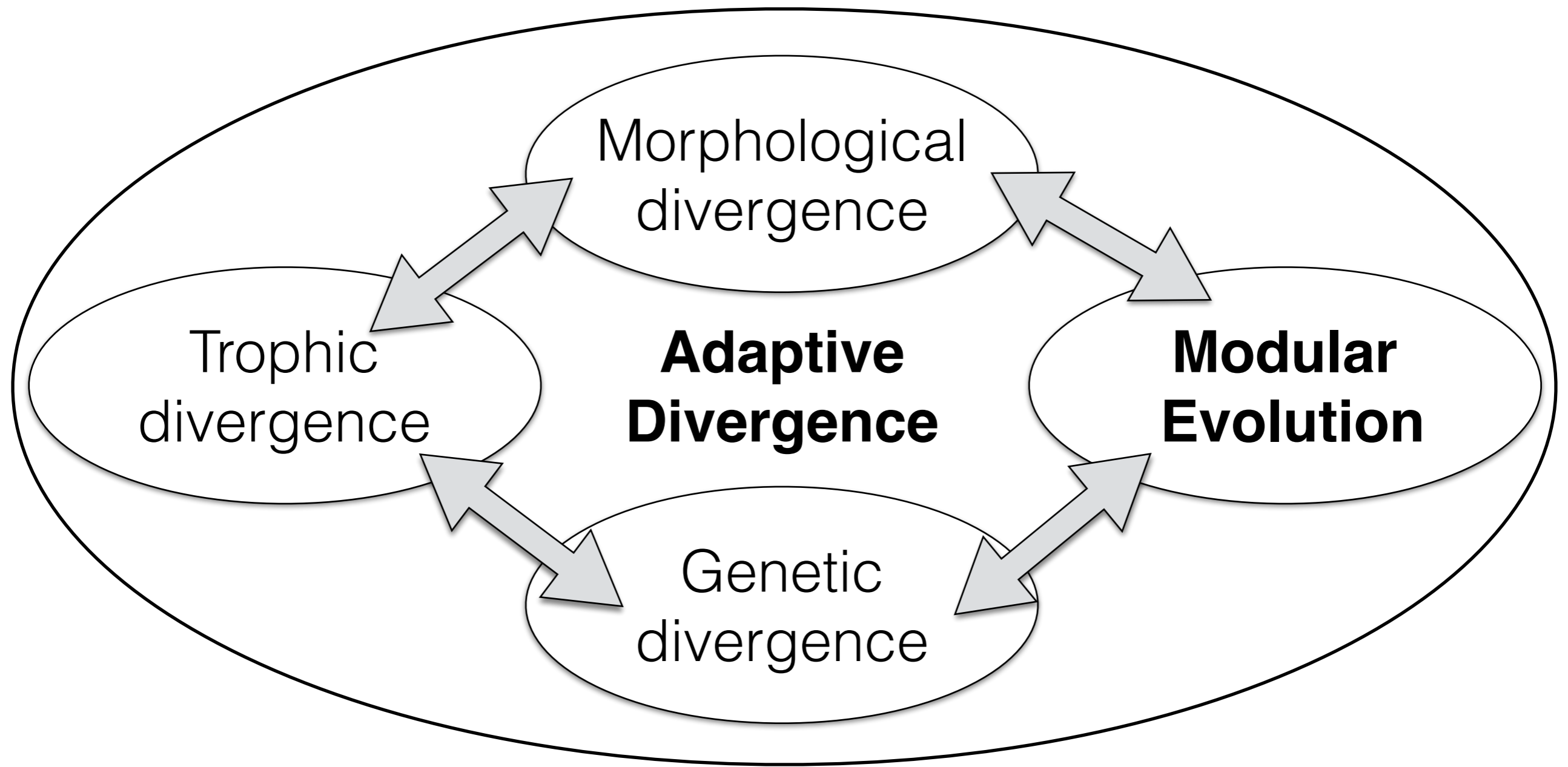

Supplement: Supplemental Information 2 — Based on this diagram we suggested a reciprocal interaction among trophic, morphological, modular and genetic divergence, which could facilitate the adaptive divergence. [file peerj-05-3851-s002.pdf]
